# The geographical distribution of lymphatic filariasis infection in Malawi

**DOI:** 10.1186/1475-2883-6-12

**Published:** 2007-11-29

**Authors:** Bagrey MM Ngwira, Phillimon Tambala, A Maria Perez, Cameron Bowie, David H Molyneux

**Affiliations:** 1 Lymphatic Filariasis Support Centre, Liverpool School of Tropical Medicine, Pembroke Place, L3 5QA, UK; 2 Malawi College of Medicine, P/Bag 360, Blantyre3, Malawi; 3 Onchocerciasis Control Programme, PO Box 2273, Blantyre, Malawi

## Abstract

Mapping distribution of lymphatic filariasis (LF) is a prerequisite for planning national elimination programmes. Results from a nation wide mapping survey for lymphatic filariasis (LF) in Malawi are presented. Thirty-five villages were sampled from 23 districts excluding three districts (Karonga, Chikwawa and Nsanje) that had already been mapped and Likoma, an Island, where access was not possible in the time frame of the survey. Antigenaemia prevalence [based on immunochromatographic card tests (ICT)] ranged from 0% to 35.9%. Villages from the western side of the country and distant from the lake tended to be of lower prevalence. The exception was a village in Mchinji district on the Malawi-Zambia border where a prevalence of 18.2% was found. In contrast villages from lake shore districts [Salima, Mangochi, Balaka and Ntcheu (Bwanje valley)] and Phalombe had prevalences of over 20%.

A national map is developed which incorporates data from surveys in Karonga, Chikwawa and Nsanje districts, carried out in 2000. There is a marked decline in prevalence with increasing altitude. Further analysis revealed a strong negative correlation (R^2 ^= 0.7 p < 0.001) between altitude and prevalence. These results suggest that the lake shore, Phalombe plain and the lower Shire valley will be priority areas for the Malawi LF elimination programme. Implications of these findings as regards implementing a national LF elimination programme in Malawi are discussed.

## Background

Lymphatic filariasis (LF) has been identified as a major public health problem and is endemic in over 80 countries. It is currently estimated that up to 120 million people are infected with *Wuchereria bancrofti *in about 83 endemic countries [1]. Of these, it is estimated 40 million people have evidence of chronic manifestations such as hydrocele and lymphoedema/elephantiasis. In addition the affected individuals suffer repeated episodes of adenolymphangitis ('acute attacks') which result in marked loss in their economic productivity [2]. Improved therapies and diagnostic methods have led to the realisation that it should be possible to interrupt transmission and eliminate LF by repeated, annual cycles of mass drug administration (MDA), with single dose combination regimens [3]. Thus, in 1997 the World Health Assembly passed a resolution calling for strengthening of activities leading to the elimination of LF as a "public health problem [4]." This resulted in the initiation of the now well-established Global Program to Eliminate Lymphatic Filariasis (GPELF) in 2000.

Malawi has two previously known LF foci: one in the southern part (Shire valley) and the other in the northern region along the Songwe river which forms its border with Tanzania [5,6]. However there had been no detailed community based surveys for LF in Malawi apart from one in the northern focus which was conducted in 1960. This survey, based on microscopic examination (for microfilarae) of thick bloodsmears which were made from samples collected at night, showed a high prevalence of microfilaraemia amongst adults (40%) and suggested that human infection with *W. bancrofti *was confined to communities in close proximity to the Songwe River [7].

More recently, surveys in these two foci have reported high antigenaemia prevalence based on immunochromatographic (ICT) card tests that approached 80% in some of the sampled villages [8,9]. There was also a higher than expected prevalence of LF associated disease in both areas (4% lymphoedema and up to 18% hydrocele). In addition, the survey in Karonga established that *W. bancrofti *infection is more wide spread than previously recognised, whereas in the lower Shire valley a markedly higher antigenaemia prevalence (55%) was found amongst children (aged 1–9 years) than what has been reported in any of the published literature.

Towards the end of 2003 we completed a nation-wide mapping exercise using ICT cards. The objective was to obtain data on the geographical distribution of LF in the remaining districts in Malawi as a prerequisite to initiating national LF elimination activities. This paper presents findings from a 2003 survey and incorporates data from recent surveys in the two known foci that have already appeared in the scientific literature to produce, for the first time, a complete map of the distribution of LF infection (based on adult worm antigenaemia) in Malawi. The implications of this distribution for LF control programme planning and eventual implementation are discussed.

## Methods

Malawi is administratively divided into northern, central and southern regions. These are further divided into 28 districts. Two new districts (Neno – parent district – Mwanza and Likoma Island – parent district – Nkhata-Bay) were formed after this survey had already been planned and thus were mapped within there parent districts. In addition, access to Likoma, an Island District, was not possible in the time frame of this survey. LF prevalence data were available for three districts; Karonga District in the northern region, Chikwawa and Nsanje Districts in the southern region. The latest survey did not cover these districts. In the remaining districts we aimed to sample a random selection of villages for antigen testing. A database of villages by district was made available via the WHO's HealthMapper software. A programme incorporated in the software was used to provide a random sample of villages to be surveyed. The selected villages had a 50 km buffer zone as recommended by the WHO's rapid assessment for the geographical distribution of lymphatic filariasis (RAGFIL) method [10]. Three additional villages were chosen in the field from inhabited areas from where the database did not contain any villages. The testing protocol adopted followed recommendations of the RAGFIL method that is based on Lot Quality Sampling (LQAS) scheme [11]. Briefly, if at least 10 (20%) of the first 50 individuals (aged >15 years) tested were positive testing could be stopped; otherwise up to 100 individuals were to be tested per sampling point [11]. However since many villages are sparsely populated an adjacent village to the randomly selected one were also invited to participate in order to achieve the required sample size. Hence random selection of subjects was not feasible in most villages. Before testing could be carried out a meeting with village members was held and the objectives of the survey were explained in the local language. Each consenting individual provided demographic data (age and sex) and a finger prick blood sample. The whole blood obtained was immediately applied onto the ICT (Binax Inc., Portland, ME) card and read within ten minutes according to the manufacturer's instructions. If two lines appeared in the viewing window that particular individual was regarded as positive for LF [12]. Individuals found positive were treated on the spot with albendazole (400 mg) and ivermectin (200 μg/kg body weight). All sampled villages had geo-coordinates determined by a portable Geographical Positioning System (GPS-Garmin eTrex^®^) machine.

### Ethics

The survey received ethical clearance from the Malawi Ministry of Health Sciences Research Committee (HSRC) and from the Liverpool School of Tropical Medicine Ethics Committee. Individual consent was obtained from each participant or (if they were aged <16) from one of their parents or a guardian.

### Data Management

Data were entered into the computer using EPINFO 2000 (CDC, Atlanta) software. The data were subsequently exported into STATA version 7 (Stata Corporation, College Station, TX) for descriptive statistical analyses. In order to investigate the relationship between prevalence and altitude, log transformation of the prevalence data was carried out using the formula log_10 _(x + 1). Village geographical coordinate data were used to produce a map showing the spatial distribution of LF infection using the WHO's HealthMapper software.

## Results

**Figure 1:**
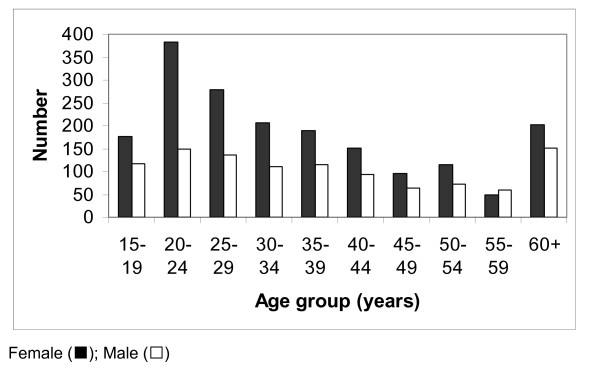
The age and sex distribution of survey participants.

**Figure 2:**
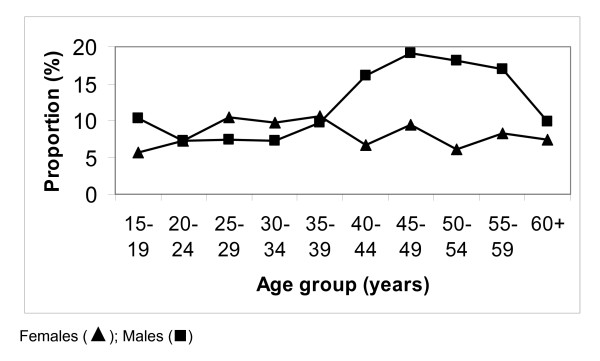
The proportion of males and females positive for CFA by age.

A total of 35 data points were sampled. Of these three were chosen in the field in inhabited areas where there were no villages on the Healthmapper database. A total of 2913 individuals were examined. The age and sex distribution of the survey participants is shown in Figure 1. There was a female excess (64%) amongst the study participants (more marked in the 20–24 age bracket). Overall there were 269 (9.2%) individuals positive for circulating filarial antigen (CFA) based on ICT results. Significantly more males than females tested positive (11.0% vs 8.2% p = 0.01). Figure 2 shows the proportion of those positive for CFA by age and sex. Amongst the males, those positive, tended to be older (student *t test *p = 0.08). This relationship was not observed in their female counterparts.

**Figure 3:**
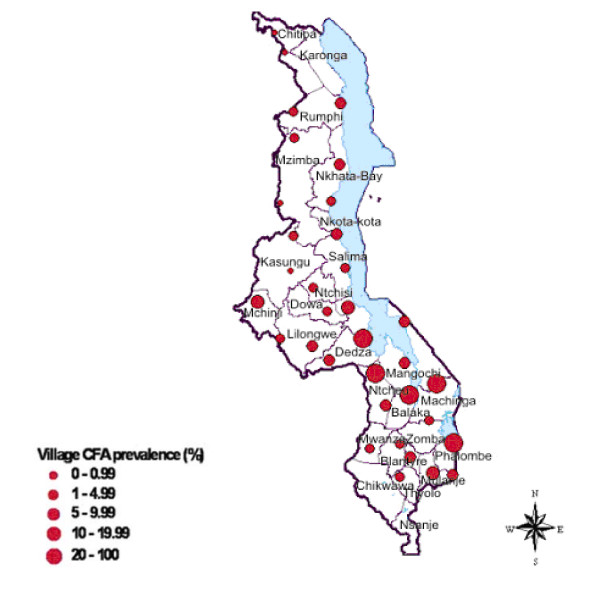
Map of Malawi showing the prevalence levels recorded in the 2003 survey.

Survey prevalence data by district and village are presented in Table 1. This ranged from 0% to 35.9%. The spatial distribution of the sampled villages with their prevalence category are shown in Figure 3. In general villages in the western side of the country registered a CFA prevalence of less than 10%. This is with the exception of Mzenga Village in Mchinji District along the Malawi-Zambia border where a prevalence of 18.2% was found. Prevalence of over 20% was observed from villages in Salima and Mangochi Districts along the southern shore of Lake Malawi. Also in Ntcheu district (Bwanje Valley), Balaka district near Lake Malombe and finally in Phalombe district along the shores of Lake Chilwa. The highest prevalence (35.9%) was recorded at Kalembo village in Balaka district in southern Malawi.

**Table 1:** ICT antigen prevalence data from the nation wide survey conducted in 2003

**District**	**Village**	**Number tested**	**Number positive**	**Prevalence**	**Latitude**	**Longitude**
Balaka	Kalembo	53	19	35.8	14.84500	35.16900
Blantyre	Masanjala Lilangwe	77	5	6.5	15.54490	35.02184
Chiradzulu	Mbalame	81	6	7.4	15.70000	35.10000
Chitipa	Chisenga	85	0	0	9.97500	33.38977
Chitipa	Siyombwe	77	0	0	9.68441	33.24764
Dedza	Kamenyagwaza	64	5	7.8	14.40750	34.98750
Dowa	Chimangamsasa	72	4	5.6	13.70964	33.99795
Kasungu	Kadyaka	65	0	0	13.07633	33.48360
Kasungu	Kaluluma	105	3	2.9	12.58077	33.51870
Lilongwe	Mwenda 1 T/A Chadza	84	6	7.1	14.14074	33.78825
Machinga	Phuteya	70	3	4.3	15.19000	35.09887
Mangochi	Chilawe	92	9	9.8	13.80000	35.10300
Mangochi	Chiponde	90	12	13.3	14.38300	35.10000
Mangochi	Mtuwa	82	21	25.6	14.68400	35.55100
Mchinji	Chalaswa	98	4	4.1	14.11689	33.32919
Mchinji	Mzenga	99	18	18.2	13.60427	32.73460
Mulanje	Gawani	78	6	7.7	15.98100	35.78300
Mulanje	Mbewa	69	13	18.8	15.99970	35.48611
Mwanza	Chapita A	64	3	4.7	15.63022	34.59139
Mzimba	Milingo-Jere	101	0	0	12.20374	33.33340
Mzimba	Kambombo	102	2	1.9	11.17551	33.52649
Nkhata-Bay	Kalumpha	104	7	6.7	12.08733	34.05695
Nkhata-Bay	Mizimu	103	8	7.8	11.55820	34.18150
Nkhotakota	Mowe	122	11	9	12.55496	34.13366
Nkhotakota	Tandwe	81	3	3.7	13.02981	34.26246
Ntcheu	Gwaza	92	26	28.3	14.52800	34.68000
Ntcheu	Nkonde-1	66	6	9.1	14.98570	34.82825
Ntchisi	Kalulu	99	3	3	13.33129	33.74804
Phalombe	Maguda	78	19	24.4	15.51774	35.78996
Rumphi	Bongololo	72	1	1.4	10.81276	33.52233
Rumphi	Mhango	82	8	9.8	10.81000	33.52379
Salima	Chipoka-Nkwizi	73	16	21.9	14.03676	34.50614
Salima	Kasonda	78	13	16.7	13.59828	34.29268
Thyolo	Nkaombe	95	6	6.3	15.99271	35.04998
Zomba	Kapenda	57	2	3.5	15.35885	35.40305

**Figure 4:**
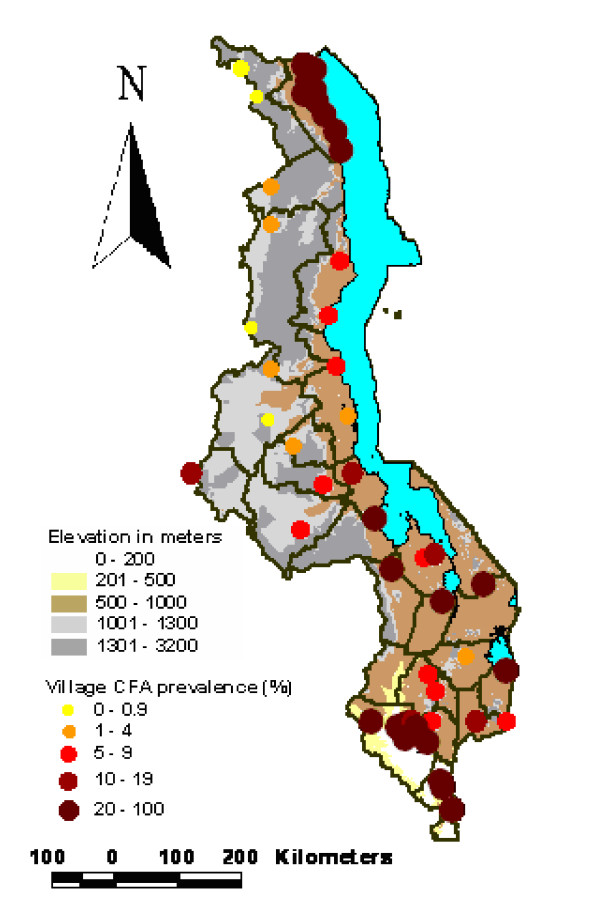
Map of Malawi showing prevalence of all sampled villages (except 2 villages in Nsanje District) in relation to altitude (metres).

**Figure 5:**
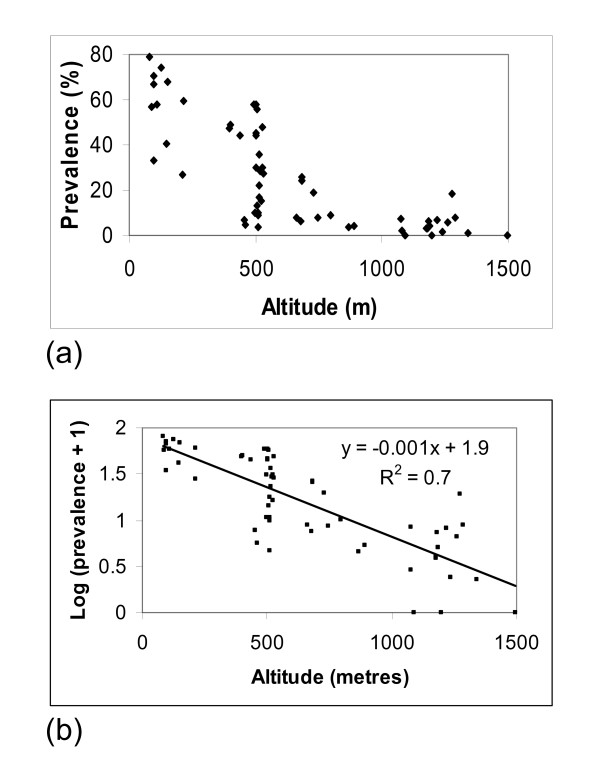
Prevalence plotted against altitude (metres) (a) and log transformed (prevalence + 1) plotted against altitude (metres) (b).

Prevalence data from the 2000 surveys are summarised in Table 2. The geographical distribution of data points sampled (ICT) in Malawi (except two villages in Nsanje District where it was not possible to obtain geographical coordinates) showing prevalence in relation to altitude is presented in Figure 4. Figure 5(a) shows a scatter plot of antigen prevalence by altitude. There is notable decline in prevalence with increasing altitude and further statistical analyses on log transformed prevalence data [Figure 5(b)] have shown a significant negative correlation between altitude and prevalence (R^2 ^= 0.7 p < 0.001).

**Table 2:** ICT antigen prevalence data from surveys conducted in 2000

**District**	**Village**	**Number tested**	**Number positive**	**Prevalence**	**Latitude**	**Longitude**
Karonga	Mwenitete	42	20	47.6	9.71257	33.92973
Karonga	Mwakyusa	91	44	48.7	9.69795	33.89313
Karonga	Mwenepela	102	59	57.8	9.67193	33.8252
Karonga	Kashata	50	22	44	9.73315	33.88652
Karonga	Mwamsaku	50	22	44	9.8092	33.86483
Karonga	Mwambetania	50	29	58	9.86747	33.86892
Karonga	Kafikisila	51	23	45.1	9.91213	33.93105
Karonga	Mwenitete-mpata	50	24	48	9.94957	33.82237
Karonga	Ngosi	50	15	30	10.01228	33.94907
Karonga	Mwakabanga	50	15	30	10.14422	34.01782
Karonga	Kanyuka	51	14	27.5	10.30768	34.12692
Karonga	Bonje	50	28	56	10.49027	34.17098
Nsanje	Chazuka	148	60	40.5	16.84261	35.25259
Nsanje	Nchacha18	148	86	58.1	16.63617	35.17126
Nsanje	Gamba	84	56	66.7	16.5811	35.14076
Chikwawa	Nchingula	128	76	59.4	15.99828	34.48297
Chikwawa	Zilipaine	129	96	74.4	16.07998	34.88262
Chikwawa	Mbande	108	76	70.4	16.16167	34.79332
Chikwawa	Pende	116	79	68.1	16.04362	34.72428
Chikwawa	Belo	196	155	79.1	16.02093	34.8162
Chikwawa	Mfunde	87	29	33.3	16.19929	35.01652
Chikwawa	Kasokeza	60	34	56.7	16.11213	34.92532
Chikwawa	Khumbulani	59	9	15.3	15.99232	34.8791
Chikwawa	Muyaya	78	21	26.9	16.04667	34.90783

## Discussion

The present survey, in the remaining unmapped districts in Malawi, has shown that infection with *W. bancrofti *as determined by antigenaemia prevalence is more widespread than previously appreciated. The female excess observed amongst our survey population probably reflects the fact that males are often out in the field during the day thus not available for testing. The implication of this being that the prevalence we found in some of our sampled villages is likely to be an under-estimate of the true prevalence. This is due to the fact that in most communities significantly more males tend to carry the infection as has been observed in this survey and in other surveys from Malawi and elsewhere in Africa [9,13].

In all districts, except Chitipa in the north, there was at least one individual who was positive on ICT. The low prevalence found in villages from the western side of Malawi could be explained by the fact that these areas are dry, of relatively higher altitude and thus not ideal for extensive mosquito breeding. The 18.2% prevalence observed at Mzenga Village in Mchinji along the Zambia border is intriguing. This is particularly so as there have been no anecdotal reports of LF disease from either the Malawi or Zambia side of the border in this area. Of note is that this village is in close proximity to a perennial stream that sustains a reasonable amount of irrigated onion farming. Whether this setting is conducive for supporting extensive mosquito breeding and thus driving *W. bancrofti *infection as has been observed in Northern Malawi and Ghana will need further investigation [14]. Ideally this should be coupled with human night blood examination for microfilariae.

It is also interesting to note that some villages from districts (Rumphi, Nkhata-Bay and Nkhotakota) along the lake shore had prevalence of less than 10%. A possible explanation could be due to the fact that these districts are mountainous and thus well drained consequently limiting potential mosquito breeding sites.

The relatively high prevalence found in Salima, Ntcheu (Bwanje Valley), Balaka, Mangochi and Phalombe was unexpected. However there have been isolated unpublished reports of cases with chronic manifestation of LF (hydrocele and elephantiasis) in these areas. It is worth noting that the ecological conditions in these districts are ideal for supporting large potential LF vector populations. Incorporating data from 2000 surveys clearly shows that the priority areas for LF control activities in Malawi will be the lakeshore districts, Phalombe plain and the Lower Shire Valley.

The decline in LF prevalence with increasing altitude has also been reported from other settings in Africa [15]. This is believed to be due to the influence of altitude on temperature which is known to be critical for survival of the vector and development of the parasite within the vector [16].

These findings have important implications for initiating the "Malawi LF Elimination Programme". First, following WHO's recommendation that all implementation units with a prevalence on ICT of over 1% be considered endemic and thus treated, the Malawi programme would involve 27 districts with a target population of over ten million. The population affected is far greater than ever envisaged. Secondly, both the northern (Karonga) and Southern foci (the Lower Shire Valley) share international borders which are largely porous. This calls for innovative approaches in carrying out control activities as they have to be synchronised with those in neighbouring countries. Thirdly, in some districts (Phalombe, Mulanje, Thyolo, Chikwawa and Mwanza) where LF is co-endemic with onchocercisasis the two programmes will need to be merged. Fourthly, the LF programme will need to establish links with other programmes that are delivering community based interventions such as the ministry of education's deworming and feeding programme and the expanded bed net distribution under the malaria control programme.
